# A non-toxic analgesic elicits cell-specific genomic and epigenomic modulation by targeting the PAG brain region

**DOI:** 10.1016/j.ynpai.2025.100192

**Published:** 2025-07-20

**Authors:** Hernan A. Bazan, Brian L. Giles, Surjyadipta Bhattacharjee, Scott Edwards, Nicolas G. Bazan

**Affiliations:** a Division of Vascular and Endovascular Surgery, Sutter Health, California Pacific Medical Center, San Francisco, CA 94109, USA; b Neuroscience Center of Excellence, School of Medicine, Louisiana State University Health New Orleans, New Orleans, LA 70112, USA; c Department of Physiology, School of Medicine, Louisiana State University Health New Orleans, New Orleans, LA 70112, USA

**Keywords:** Non-opioid, Non-hepatotoxic analgesia, Single-cell multiome, Epigenomics, Transcription factors, Cell-cell interaction

## Abstract

Acetaminophen (ApAP) is widely used for pain management, but overuse or overdose leads to hepatotoxicity, making it the leading cause of acute liver failure globally. There is an urgent need for safer pain medications, as other non-opioid analgesics like non-steroidal anti-inflammatory drugs (NSAIDs) are nephrotoxic. We have identified SRP-001 as a safer, non-hepatotoxic, novel analgesic that overcomes ApAP’s limitations by avoiding NAPQI formation and preserving hepatic tight junctions. Using coupled RNA and ATAC sequencing, from the periaqueductal gray (PAG) midbrain region, we compared the genetic and epigenetic signatures of SRP-001 and ApAP treatments following complete Freund’s adjuvant (CFA)-induced inflammatory pain against no treatment and vehicle controls. Our analysis revealed differential activity in three transcription factor families (SOX, SP/KLF, and AP-1) with cell-specific patterns and altered neuron-neuron interactions through neurexin-neuregulin signaling. SRP-001 and ApAP demonstrated similar genetic and epigenetic outcomes, indicating that SRP-001 is a favorable alternative due to its non-hepatotoxic properties while maintaining the same antinociceptive effects as ApAP.

## Highlights

• SRP-001 generates AM404 in PAG, activating TRPV1 for central analgesia, relieving pain without hepatotoxicity.
• SRP-001 restores SOX in oligodendrocytes and SP/KLF in neurons, supporting myelination and neural repair during chronic pain.
• SRP-001 epigenetically inhibits AP-1 and TFEB transcription factors, promoting anti-inflammatory effects without gene changes.
• SRP-001 restores Neurexin-Neuroligin synaptic signaling, protecting neurons and synaptic integrity in inflammatory pain.

## Introduction

Acute and chronic pain management is one of the most prevalent and costly public health issues worldwide. In the United States alone, pain affects more adults than heart disease, diabetes and cancer combined (Institute of Medicine (US) Committee on Advancing Pain Research), imposing an estimated annual cost of $635 billion on the healthcare system (Gaskin and Richard, 2012).

Current medications either carry a risk of substance use disorders – such as opioids – or cause organ damage with overuse, like liver toxicity from acetaminophen (ApAP) or kidney harm from non-steroidal anti-inflammatory drugs (NSAIDs). The use of narcotics for pain management is closely linked to opioid use disorder (OUD) due to their high potential for abusability of this medication class. Indeed, the high levels of OUD, misuse of both illicit and prescription opioids, and opioid overdose risk in the United States underscore the urgent need for safe and effective non-opioid analgesics. The widespread prevalence of OUD, along with the misuse of both illicit and prescription opioids and the risk of opioid overdose in the United States, highlights the urgent need for safe and effective non-opioid analgesics. This need is particularly acute in dental practice, where opioids are commonly prescribed following procedures like wisdom tooth extractions, root canals, and surgical implants, which can produce acute or even prolonged pain. Despite a decrease in opioid prescription rates in dental practice due to increased national attention on the opioid epidemic (Okunev et al., 2021), dental care-related opioid prescriptions often serve as the first exposure to opioids for many adolescents. This initial exposure has been associated with an increased risk of continued opioid misuse and eventual diagnosis of OUD (Schroeder et al., 2019).

Indeed, a study published in the Journal of the American Medical Association found that patients aged 13 to 30 who filled an opioid prescription to treat pain after wisdom tooth extraction were three times more likely than their peers to continue using opioids (Harbaugh et al., 2018). The dental profession has made some progress in shifting away from overprescribing opioids. Still, due to the risk of toxicity of non-addictive alternative analgesics at the doses required to achieve adequate pain relief following oral surgeries, high rates of opioid prescriptions persist (Okunev et al., 2021). To further reduce opioid use and deliver safer, effective pain relief for more patients, new therapeutics are needed.

Several non-opioid pain relievers, including NSAIDs and ApAP, are commonly used alternatives for treating pain following dental and other procedures. Although efficacy varies across these options, a single dose of ApAP is as effective as an opioid/ApAP combination 2 h after administration in patients with moderate-to-severe acute extremity pain (Chang et al., 2017). Moreover, a *meta*-analysis of 82 RCTs reported that degree and duration of pain relief for dental pain were better with ApAP compared to opioids, and opioid use was associated with a higher rate of adverse events (Miroshnychenko, 2023).

Studies have demonstrated that 1,000 mg ApAP is optimal for post-oral surgery pain relief (Qi et al., 2012); however, the threshold for acute liver failure is only 4,000 mg daily (Yoon et al., 2016). Patients would reach this threshold with a dosing schedule of every six hours, and any increase in dose or frequency due to inadequate pain relief would exceed this limit. Unintentional overuse – such as receiving doses from multiple medicinal products without realizing some contain ApAP – is also a concern, especially in patients with compromised liver function, like older adults and individuals with alcohol-associated liver damage.

The danger of ApAP comes from the formation of the secondary metabolite N-acetyl-p-benzoquinoneimine (NAPQI) (Moore, 1985). We have previously demonstrated the safety of SRP-001 due to the lack of NAPQI formation compared to ApAP (Bazan et al., 2024). SRP-001 is oxidized by cytochrome P450 enzymes to a different N-acyl-p-benzoquinone imine rather than NAPQI, which accounts for the non-hepatotoxicity (Bazan et al., 2024). Unlike ApAP, SRP-001 does not cause centrilobular necrosis or disrupt tight junctions between hepatocytes in the liver (Bazan et al., 2024, Gamal et al., 2017). Thus, SRP-001 is a safer option for analgesic use as it prevents severe liver damage caused by ApAP chronic overuse or overdose.

Our previous study also demonstrated the antinociceptive efficacy of SRP-001 compared to ApAP (Bazan et al., 2024), although the neurobiological mechanism of action remains to be elucidated. In that publication, we comprehensively reported detailed analgesic activity across multiple preclinical pain models (CFA-induced inflammatory pain with von Frey and Hargreaves assays in Sprague-Dawley rats; tail-flick and abdominal writhing assays in CD1 and C57BL/6 mice) – demonstrated in Fig. 2, Figs. S3–S7, and Table S1). Three *in vivo* preclinical models were used – a) **Inflammatory Pain:** Complete Freund's Adjuvant (CFA)-induced inflammation in Sprague-Dawley rats, assessed using electronic von Frey (eVF) for mechanical sensitivity and Hargreaves test for thermal sensitivity, b) **Somatic Pain:** Tail flick assay in CD1 and C57BL/6 mice, measuring tail withdrawal latency to cold stimulation, and c) **Visceral Pain:** Acetic acid-induced abdominal writhing in CD1 and C57BL/6 mice, counting abdominal contractions. SRP-001 and ApAP were administered orally at 32 mg/kg and 100 mg/kg doses. A vehicle control was also used. The study included mixed-gender and varied-age rodents (young: 2 months; aged: 20 months) to assess potential gender and age-related effects. However, aged cohorts were exclusively male due to availability at commercial vendors and the National Institute on Aging (NIA). Experiments were conducted with investigator blinding to ensure objective results. The eVF device further minimized bias in mechanical sensitivity measurements. GraphPad Prism Version 9.1.2 was used for statistical analysis, with significance defined as p < 0.05 (one-way ANOVA followed by Sidak's multiple comparisons post hoc test). Equimolar (µmol/kg) ED50 values were determined from dose-response curves using regression analysis and log transformation. The experimental design adhered to the 3Rs principles (Replacement, Reduction, and Refinement) (Vitale and Ricceri, 2022, Tannenbaum and Bennett, 2015, Lewis, 2019, Verderio et al., 2023).

SRP-001 demonstrated comparable antinociceptive (pain-relieving) effects to ApAP across all tested models in both mice and rats. In the CFA model, both SRP-001 and ApAP dose-dependently increased the paw withdrawal threshold in CFA-injected paws, indicating a reduction in mechanical hypersensitivity. Similar results were observed across young male, young female, and aged cohorts. SRP-001 also increased tail withdrawal time in the tail flick assay and reduced writhes in the abdominal writhing assay, similar to ApAP. Equimolar ED50 values indicated that SRP-001 either matched or improved upon ApAP's efficacy across all antinociception models. SRP-001 also showed similar antipyretic (fever-reducing) effects to ApAP in LPS and baker's yeast fever induction models. In that study, we further investigated SRP-001′s uptake and effect in the periaqueductal gray (PAG) area of the midbrain, a crucial region for pain modulation. SRP-001 was confirmed to be present in the PAG. Remarkably, SRP-001 treatment resulted in the highest production of N-arachidonoylaminophenol (AM404) in the PAG, particularly in animals co-treated with CFA. AM404 is a known metabolite of ApAP that mediates antinociception through TRPV1 and cannabinoid 1 receptors. This suggests a potential shared or enhanced mechanism of action. While different pain assays were conducted in different rodent models (rats for von Frey/Hargreaves; mice for tail flick/abdominal writhing), we observed comparable analgesic effects of both SRP-001 and ApAP across species and strains, with SRP-001 often performing better than ApAP.

To investigate the neurobiological mechanism of action (MOA), we focused on activity within the midbrain PAG, which is known to be involved in endogenous analgesia and is the active brain site for ApAP function (Barriere et al., 2020).

To uncover the epigenetic and genomic landscape modulated by treatment with the analgesics SRP-001 or ApAP, we conducted single-cell RNA sequencing for gene expression and chromatin accessibility using the 10x Genomics Multiome platform under four conditions: saline (vehicle) treatment, CFA-induced pain exposure, CFA with SRP-001 treatment, and CFA and ApAP treatment. Midbrain PAG region was dissected from the brains of young male Sprague Dawley rats, n = 1 per condition.

## Results

Using single-cell RNA sequencing (scRNA-seq) of the PAG, we previously demonstrated that SRP-001 and ApAP share common mechanisms for pain modulation (Bazan et al., 2024). Summary of these key findings are: *FAAH-dependent endocannabinoid signaling*. SRP-001 and ApAP modulate overlapping gene networks involved in endocannabinoid signaling and mechanical nociception, particularly through FAAH inhibition. Both compounds downregulate FAAH, increasing levels of anandamide (AEA) and other antinociceptive lipids, enhancing CB1/CB2 receptor-mediated analgesia. *TRPV1 activation*. ApAP’s metabolite AM404 activates TRPV1 in glutamatergic neurons of the vlPAG, increasing glutamate release and mGlu5 receptor activity. TRPV1 plays a dual role in central and peripheral pain processing. Its activation in the PAG-RVM pathway is essential for ApAP’s analgesic effects and is a promising target for integrated pain therapies. *Ion channel regulation*. Both drugs modulate expression of key ion channels (e.g., TRPV4, KCNA1, KCNT1), suggesting a broad mechanism involving reduced neuronal excitability. Beyond calcium and sodium channels, potassium channels (e.g., K-ATP, KCa, K2P) are emerging as critical targets (Zemel et al., 2018, Beich et al., 2025, Gada and Plant, 2019). SRP-001 affects multiple ion channels, suggesting a broad inhibitory effect on neuronal excitability. We expand on these findings by integrating single-cell ATAC-seq data, providing novel genomic and epigenomic insights into pain modulation. This multi-omic approach reveals regulatory elements and chromatin accessibility patterns that underlie the transcriptional changes observed previously.

### Cellular architecture of PAG samples by single-nuclei RNA sequencing

We isolated cells from each condition – Vehicle, CFA, SRP-001, and ApAP – and sequenced them. Aggregated filtered feature matrices and fragment outputs for Multiome Gene Expression (GEX) and Assay for Transposase Accessible Chromatin (ATAC) from CellRanger- ARC pipeline (10x genomics) were exported for analysis using Seurat (Vaccarino et al., 2007). Following quality control within Seurat, we obtained 5,658 cells in the Vehicle, 4,722 cells in the CFA, 7,991 cells in the SRP-001, and 2,017 cells in the ApAP condition for further analysis. There were no notable differences in any quality control measurements between conditions (Fig. S1A). Dimensionality reduction was performed for all cells and neuronal cluster subset using PCA for GEX and LSI for ATAC (Fig. S1B). Clustering was performed using and visualized using Uniform Manifold Approximation and Projections (UMAP) plots. GEX and ATAC data were integrated using weighted-nearest neighbor (WNN) analysis (Hao et al., 2021). Integrated UMAPs displayed no batch effects (Fig. S2A) and yielded 33 distinct clusters across all cell types. Neuronal subset underwent same analysis and yielded 34 distinct clusters in neuronal subset (Fig. S2B).

**Fig. 1:**
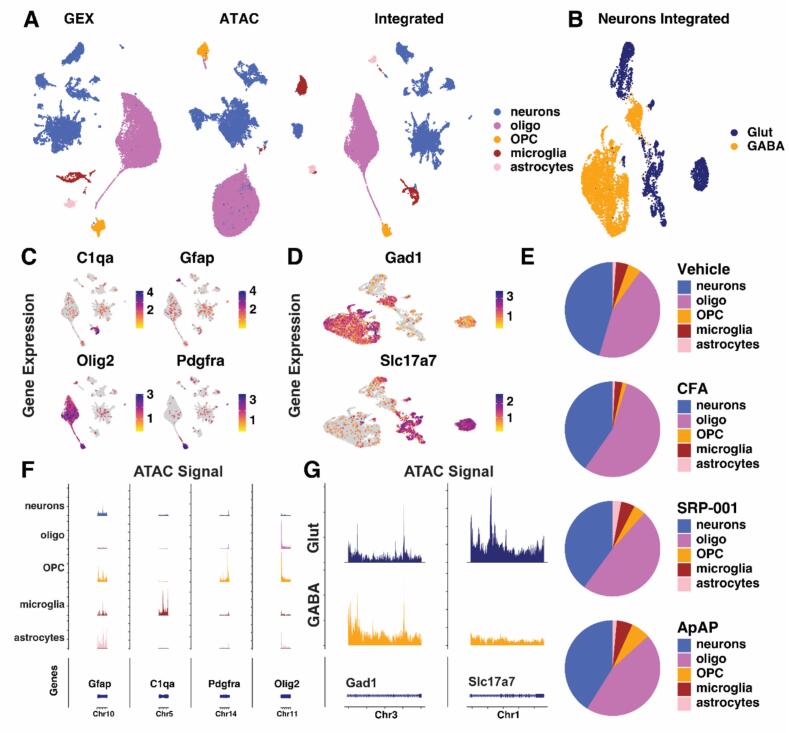
Cell Clustering and Annotation. UMAPs by RNA, ATAC, and Integrated by Weighted Nearest Neighbor Analysis (WNN) with Cell Type Annotation (A). WNN UMAP of Neuron Subset with Neuronal Cell Type Annotation (B). Cell Marker Feature Plot for Non-Neuronal Cell Types (C) and Neuronal Cell Types (D). Pie Charts for each sample display proportions of annotated cell types (E). Cell Marker Coverage Plot for Non-Neuronal Cell Types (F). Cell Marker Coverage Plot for Neuronal Cell Types (G).

Following clustering, five distinct cell types were identified: oligodendrocytes (oligos), oligodendrocyte precursor cells (OPCs), microglia, astrocytes, and neurons based on known marker genes. These genes include Olig2 for oligos, Pdgfra for OPCs, C1qa for microglia, and Gfap for astrocytes, and were visualized using FeaturePlot for GEX data (Fig. 1C) and CoveragePlot for ATAC data (Fig. 1F). Subsequent annotation within neuron subcluster differentiated them into two separate clusters (Fig. 1B): glutamatergic neurons (Glut) and GABAergic neurons (GABA). Annotations were validated using Slc17a7 for Glut and Gad1 for GABA in the GEX data (Fig. 1D) and ATAC data (Fig. 1G).

**Fig. 2:**
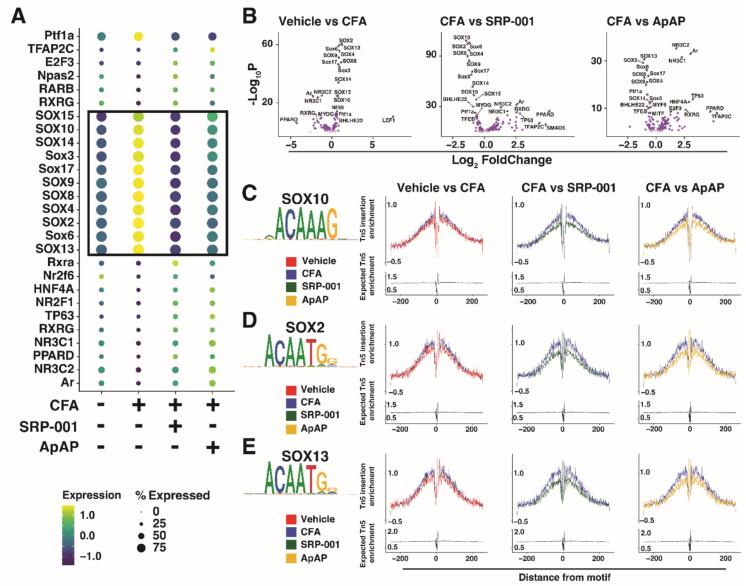
Oligodendrocyte Motif Enrichment Analysis. Dot Plot of top enriched transcription factor (TF) motifs between conditions in oligodendrocytes (A). Volcano Plots of motif enrichment via ChromVAR in Oligodendrocytes for Saline Vehicle vs CFA Vehicle, CFA Vehicle vs CFA 3DDA, and CFA Vehicle vs CFA APAP (B). TF Footprints between conditions with consensus sequence for SOX10 (C), SOX2 (D), and SOX13 (E).

Cell type proportions were determined for each condition based on cell count (Tables S1 and S2) and were visualized using pie charts (Fig. 1E). An increase in the proportion of oligos was observed in CFA (55 %) compared to SRP-001 (48 %), ApAP (46 %) and Vehicle-treated animals (44 %). There was also an increase in the proportion of OPCs in the SRP-001 (4 %) and ApAP (7 %) treatment conditions compared to CFA (1 %); however, these counts are very low overall. Similarly, there is an increase in the proportion of microglia in the SRP-001 (5 %) and ApAP (5 %) treatment conditions compared to CFA (2.5 %), but again, cell counts are low. Astrocyte composition was around 1 % in Vehicle, CFA, and ApAP conditions and 3 % in the SRP-001 condition. Within neuronal clusters, there was a decrease in the proportion of Glut neurons in CFA (41 %), SRP-001 (35 %), and ApAP (30 %) conditions compared to Vehicle (45 %), along with an increase in GABA neurons in CFA (59 %), SRP-001 (65 %), and ApAP (70 %) conditions compared to Vehicle (55 %).

### SOX TF activation by CFA, recovery with treatments

Transcription factor (TF) activity within each cell was determined using chromVAR (Mistry, et al., 2023) and revealed an increase in activity of SOX family TFs in Oligos of CFA compared to Vehicle, SRP-001, and ApAP. ChromVAR allows for the measurement of specific TF associated chromatin accessibility by measuring the relative accessibility at peaks across all motifs corresponding with the TF binding site along each chromosome of each cell. This technique is powerful as it is able to accurately infer TF activity in each individual cell and can be used to compare between conditions while still maintaining cell-type specificity. From chromVAR outputs, the top motif accessibility changes between all conditions were normalized and plotted on a dotplot (Fig. 2A). Notably, SOX family TFs displayed the largest accessibility changes between conditions, with an increase in accessibility and thus an increase in SOX TF activity in CFA compared to other conditions. Individual comparisons between Vehicle vs CFA, CFA vs SRP-001 and CFA vs ApAP were visualized as volcano plots (Fig. 2B). Between Vehicle and CFA, there is a clear increase in SOX TFs and decrease in NR3C2, NR3C1, and Ar in CFA. Between CFA and SRP-001, as well as between CFA and ApAP, there is a decrease of SOX TFs and an increase in NR3C2, NR3C1, and Ar in analgesic groups compared to CFA. This observation indicates that CFA-induced pain results in the increased activity of SOX TFs and decreased activity of NR3C2, NR3C1, and Ar TFs, but with analgesic treatment, the activation returns closer to baseline as the activity is decreased in SOX TFs and increased in NR3C2, NR3C1, and Ar. The top deviated SOX TFs were plotted using TF Footprint to map activity to specific consensus sequence motifs for TF binding. Footprints measure TF binding by analyzing disruptions in Tn5 transposase activity in ATAC sequencing. TF binding to DNA protects the protein-DNA binding site from transposition, resulting in low DNA accessibility at the motif site and high accessibility in the immediate flanking sequence (Corces et al., 2018). Thus, increased Tn5 enrichment at the motif site indicates increased TF binding activity.

This increase was observed for SOX10 (Fig. 2C), SOX2 (Fig. 2D), and SOX13 (Fig. 2E) in CFA condition compared to Vehicle, SRP-001, and ApAP conditions. From this analysis, it appears that the activity of SOX family TFs is induced by inflammatory pain conditions and that SRP-001 or ApAP treatment restores activity to normal levels. This was inferred by the difference between Vehicle and CFA, but no apparent difference between Vehicle and SRP-001 or ApAP conditions.

Functionally, SOX TFs are involved in stem-cell renewal and differentiation (Stevanovic et al., 2023). This function is highly relevant to oligos in which they recruit OPCs for increased abundance in the fiber tract environment of the PAG that requires consistent myelination. In the context of this study, pain is known to be associated with demyelination in the central nervous system (Chen, 2023). Thus, the recruitment of OPCs could be a compensatory brain mechanism for recovery. However, this recruitment via SOX TFs is not observed following treatment with SRP-001 or ApAP. The activity of SOX TFs in these treatment conditions closely resembles that of Vehicle (Fig. 2A), while the abundance of oligos and OPCs is nearly the same between conditions (Fig. 1E), indicating that analgesic treatment with SRP-001 or ApAP modulates oligodendrocyte recruitment and maturation independent of SOX TF family activity.

The SOX TF family has many members with associated functions. SOX10 has been linked to the recruitment and proliferation of oligodendrocytes (Pozniak, 2010) and identified as a transcriptional regulator of myelination (Li et al., 2007). Restoration of SOX10 levels to baseline with analgesic treatment supports myelination and cellular structural maintenance in oligodendrocytes. This effect, without hepatotoxic risks, underlines SRP-001′s role in promoting nerve health and recovery. SOX2 is involved in maintaining oligo proliferation during demyelination and thus has an important role in remyelination (Zhao, 2015), functioning through transcriptional regulation of genes involved in oligodendrocyte differentiation onset (Rupprecht et al., 2024). SOX2 also directly interacts with TGF-β signaling via transcriptional regulation (Tompkins et al., 2009), which is involved in oligodendrocyte differentiation (McKinnon et al., 1993) and inflammation (Yoshimura et al., 2010). SOX13 has been linked to inflammatory suppression in endothelial cells (Demos, 2022) and may have a similar role in oligodendrocytes. Decreased activity of SOX2 and SOX13 in the SRP-001- and ApAP-treatment conditions likely induces inflammatory-suppressing mechanisms independent of SOX TFs.

### SP/KLF TF restored by SRP-001 or ApAP

**Fig. 3:**
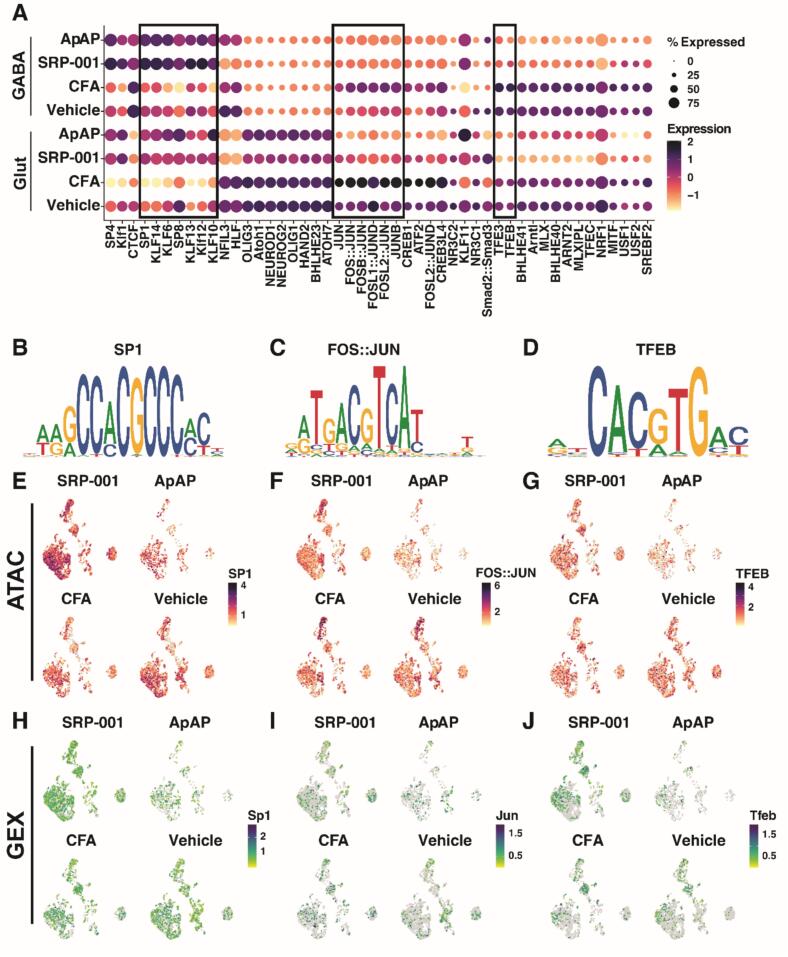
Neuron Motif Enrichment Analysis. Dot Plot of top enriched TF motifs between conditions in neurons (A). Consensus sequences for SP1 (B), FOS::JUN (C), and TFEB (D) motifs. Motif Feature Plots for SP1 (E), FOS::JUN (F), and TFEB (G). Gene Expression Feature Plots for SP1 (H), FOS::JUN (I), and TFEB (J).

TF activity analysis in neurons using chromVAR revealed a decrease in the activity of the SP/KLF family of TFs in CFA compared to Vehicle, but an increase in activity under analgesic treatment conditions compared to CFA (Fig. 3A). The treatment conditions appear to return transcription factor activity to normal levels in both Glut and GABA neurons, as the activity between Vehicle, SRP-001, and ApAP does not differ significantly. The motif activity of SP1, based on ATAC activity consensus sequence (Fig. 3B), was increased in both Glut and GABA neurons under analgesic treatment conditions compared to CFA (Fig. 3E). Despite this increase, the gene expression of Sp1 was not changed across conditions (Fig. 3H). This indicates that the decreased cellular behavior of SP1 in CFA is independent of gene expression. SP1 is involved in apoptosis regulation by maintaining neuronal survival (Ryu et al., 2003). The pro-survival role of SP1 is decreased in the context of CFA pain but recovered with treatment, indicating that treatment with SRP-001 or ApAP promotes neuronal survival mechanisms at the epigenome level, potentially offering sustained neuroprotection against aberrant pain-related plasticity. KLF TFs have been identified in the negative regulation of neurite outgrowth (Moore and Goldberg, 2010). The decrease in KLF family activity in the context of CFA indicates a compensatory mechanism for increasing neurite outgrowth. Under analgesic treatment conditions, KLF activity is similar to the activity in Vehicle, indicating that treatment with SRP-001 or ApAP may regulate neurite outgrowth independently of KLF TFs.

### AP-1 and TFEB TF deactivated by treatments

TF activity analysis in neurons also revealed two other TF families with distinct changes based on CFA pain and analgesic treatment conditions. The activity of AP-1 family TFs including FOS and JUN family TFs appears to decrease with analgesic treatment by SRP-001 or ApAP (Fig. 3A). The FOS and JUN family TF activity was measured by ATAC activity at a consensus sequence (Fig. 3C). There was no apparent difference between Vehicle and CFA, but significant decreases were observed under SRP-001 and ApAP conditions (Fig. 3F). Despite this epigenetic modulation, the gene expression of Jun (Fig. 3I) did not change between conditions, indicating a cellular behavior that is not influenced by gene expression. A similar pattern is observed with TFEB/3 TFs (Fig. 3A). There is a decrease in TF activity under SRP-001 and ApAP conditions compared to CFA and Vehicle (Fig. 3G) based on ATAC signal at a consensus sequence (Fig. 3D). Similar to AP-1 TFs, this change is independent of gene expression, as there is no change in the gene expression of Tfeb across conditions (Fig. 3J). AP-1 TF’s have been identified as displaying potential messenger activity in pain transduction and an overall role in pain signaling (Naranjo et al., 1991, Ahmad and Ismail, 2002, Hunt et al., 1987, Sonnenberg et al., 1989). The decrease observed under analgesic treatment conditions indicates that treatment by SRP-001 or ApAP confers a negative modulation of pain signaling. AP-1 TFs are also known to be activated by inflammation (Kyriakis, 1999). Within the CFA inflammatory pain model, the decrease in AP-1 TFs in treatment groups indicates that SRP-001 or ApAP promotes an anti-inflammatory response. Similarly, TFEB has a role in inflammation (Brady et al., 2018). The decrease in TFEB activity independent of gene expression under the analgesic treatment conditions also points to the potential epigenetic anti-inflammatory role of SRP-001 and ApAP. The decrease in TFEB activity independent of gene expression in the analgesic treatment groups also points to the potential epigenetic anti-inflammatory role of SRP-001 and ApAP.

### Analgesics restore neuron communication via neurexin-neuroligin

**Fig. 4:**
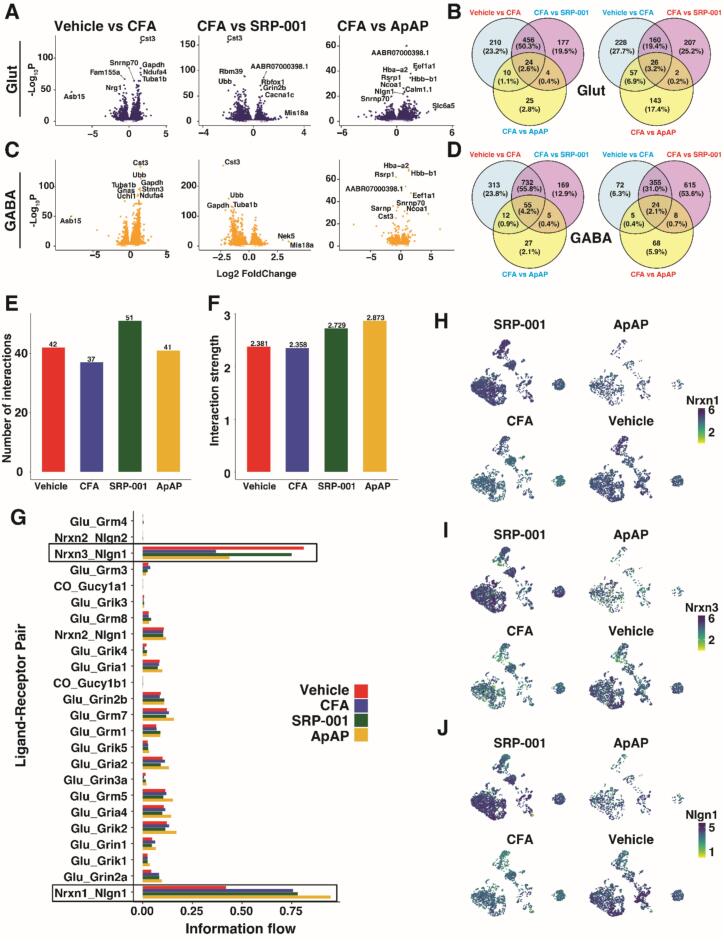
Transcriptomic Response to CFA via SRP-001 and APAP in Neurons. Volcano Plots for Differential Expression between Saline Vehicle vs CFA Vehicle, CFA Vehicle vs CFA 3DDA, and CFA Vehicle vs CFA APAP for Glutamatergic (A) and GABAergic (C) Neurons. Venn Diagrams of Overlapping Differential Expression between Saline Vehicle vs CFA Vehicle, CFA Vehicle vs CFA 3DDA, and CFA Vehicle vs CFA APAP for Glutamatergic (B) and GABAergic (D) Neurons where blue color indicates only genes going down in comparison and red indicates only genes going up in comparison. Neuron Chat analysis displaying differential communication between neurons based on number (E) and strength (F) of interactions. Bar Plot showing strength of interaction of Ligand-Receptors pairs for each condition (G). Feature Plots showing Gene Expression of genes involved in Ligand-Receptor pairs highlighted: Nrxn3 (H), Nrxn1 (I), and Nlgn1 (J). (For interpretation of the references to color in this figure legend, the reader is referred to the web version of this article.)

Differential expression testing to identify differentially expressed genes (DEGs) was conducted between conditions for each cell type to uncover genetic modulation by CFA-induced pain and analgesia treatment by SRP-001 or ApAP. Focusing on neurons, between Saline and CFA, there were 1171 significant DEGs in Glut neurons and 1586 significant DEGs in GABA neurons (pval < 0.05 after adjusting for multiple testing). Between CFA and SRP-001, there were 1065 significant DEGs in Glut neurons and 1963 significant DEGs in GABA neurons. Between CFA and ApAP, there were 291 significant DEGs in Glut neurons and 204 significant DEGs in GABA neurons (Fig. 4 *A* and *C*). Likely due to the low number of cells in ApAP (Table S1), there were notably less significant DEGs.

To better understand the genomic response in both Glut and GABA neurons caused by the analgesic treatments, the DEGs upregulated between Vehicle and Saline were compared with the DEGs downregulated between CFA and SRP-001 and CFA and ApAP, as well as the DEGs downregulated between Vehicle and CFA were compared with the genes upregulated between CFA and SRP-001 and CFA and ApAP (Fig. 4 *B* and *D*). In Glut neurons, there was a notable overlap between upregulation in CFA compared to Vehicle and downregulation in SRP-001 compared to CFA, namely a 50.5 % overlap consisting of 456 DEGs (Fig. 4B). A similar observation was made in GABA neurons, with the same comparisons having a 55.8 % overlap consisting of 732 DEGs (Fig. 4D). The overlap in DEGs downregulated in the context of CFA compared to Vehicle and upregulated in SRP-001 compared to CFA was 19.4 % consisting of 160 DEGs in Glut neurons (Fig. 4B) and 31.0 % consisting of 355 genes in GABA neurons (Fig. 4D). There was little overlap in comparisons consisting of ApAP compared to CFA, likely due to the low number of cells and thus low overlap of all 3 comparisons. However, the strong 50.3 % overlap in Glut and 55.8 % in GABA for DEGs upregulated in CFA compared to Vehicle and downregulated in SRP-001 compared to CFA indicates a strong genomic response by SRP-001 in the PAG. SRP-001 exhibits a stronger impact on DEGs in glutamatergic and GABAergic neurons than ApAP, potentially hinting at a more robust genomic response relevant to pain modulation.

To determine differences in cell–cell interactions between neurons, ligand-receptor pair (LRP) expression analysis was conducted via NeuronChat. Comparative analysis between samples revealed an increase in the number of inferred interactions between neurons in Vehicle and analgesic treatment groups compared to CFA (Fig. 4E) as well as an increase in the interaction strength in analgesic treatment groups compared to Vehicle and CFA (Fig. 4F). To determine which specific interactions played a role in the differential communication in neurons between conditions, we observed relative information flow between LRPs in the NeuronChat database which revealed differential communication in Nrxn3-Nlgn1 interactions and Nrxn1-Nlgn1 interactions (Fig. 4G). Gene expression levels were displayed in neurons to validate the differences in expression corresponding to the LRP expression analysis (Fig. 4H-J). Nrxn3-Nlgn1 was increased in Vehicle and analgesic treatment groups compared to CFA and Nrxn1-Nlgn1 was increased in CFA compared to Vehicle and further increased in analgesic treatments. This indicates that Nrxn3 and Nlgn1 interactions are decreased by pain stimuli, but analgesic treatment causes recovery, while Nrxn1 and Nlgn1 interactions are promoted by pain stimuli and maintained and potentially increased by analgesic treatments. Neuregulin-Neurexin signaling is well-known to be involved in synaptic development and synaptic maintenance (Craig and Kang, 2007, Südhof, 2008). The decrease in Neuregulin-Neurexin signaling under CFA compared to Vehicle conditions indicates a decrease in synapse maintenance or new synapse formation with pain. Analgesic treatment groups do not have the gene expression decrease in Neuregulin-Neurexin signaling, indicating that synapse disruption caused by pain is corrected with analgesic treatment at the genome level. SRP-001 shows a more pronounced recovery in this pathway, which is critical for sustaining long-term neural health and supporting resilience against pain. SRP-001 and ApAP treatment appear to be involved in the genetic recovery of Neurexin’s and Neuregulin’s for the maintenance of synaptic integrity, stability, plasticity, and downstream signaling during pain.

### Overview of genetic and epigenetic changes across conditions

Differential expression analysis for GEX data was performed for each cell type to determine genetic changes between conditions and can be found in Fig. S3-4 as well Data S1. The differences in the epigenetic landscape of each condition were determined by differential accessibility (DA) on the ATAC data to reveal differentially accessible regions (DARs) between conditions. In GABA neurons, there were 204 DARs between Vehicle and CFA, 621 DARs between CFA and SRP-001, and 85 DARs between CFA and ApAP conditions. In Glut neurons, there were 23 DARs between Vehicle and CFA, 70 DARs between CFA and SRP-001, and 11 DARs between CFA and ApAP conditions (pval < 0.05 after adjusting for multiple testing). Like the GEX data, we see a smaller response in ApAP, but this is likely due to a lower cell count causing a decrease in statistical power. There appears to be a stronger response in GABA neurons with SRP-001 treatment, highlighting analgesic engagement in pathways critical to pain relief. Top DAR for Glut neurons, GABA neurons, and oligos can be found in Figs. S5-S7, respectively. DA outputs and well as ChromVAR outputs for each cell type can be found in Data S2 and Data S3.

## Discussion

Pain and its management pose huge unmet public health challenges due to the lack of safety in existing analgesics such as NSAIDs, opioids, and ApAP. Small dosages of ApAP are commonly considered safe due to the low therapeutic index of ApAP, but overuse can lead to pronounced hepatotoxicity, leading to acute liver failure and increased mortality. Additionally, NSAIDs can cause nephrotoxicity (kidney toxicity). Liver and kidney toxicity is increased in infants, the elderly, and individuals with compromised liver and kidney function, highlighting the need for safer and inclusive analgesic options. Furthermore, chronic ApAP use is linked with liver toxicity and exacerbation of ApAP toxicity by alcohol drinkers. These individuals are also susceptible to kidney toxicity, and there is a risk of chronic renal insufficiency progressing to dysfunction and even dialysis with NSAID overuse. Thus, there is a need for new pain-relieving compounds with greater efficacy, less hepatotoxicity, and less nephrotoxicity for use in broad populations.

In place of the hepatotoxic ApAP, nephrotoxic NSAIDs, and addictive opioids, we have developed a safe, non-hepatotoxic and non-addictive alternative by eliminating the formation of the damaging secondary metabolite NAPQI. Our previous study demonstrated the efficacy of SRP-001 along with the lack of NAPQI and maintenance of hepatic tight junction stability while beginning to uncover the genetic response to SRP-001 in the midbrain PAG ^11^. To expand on that study, we dove into genomic and epigenomic modulation by SRP-001 and ApAP to better understand the mechanism of action of both analgesic options.

We discovered a consistent response at the genome and epigenome level with both analgesic treatments in the context of CFA-induced pain, indicating that the hepatotoxicity caused by ApAP can be eliminated while still maintaining crucial analgesic function in the newly developed SRP-001. Neither CFA pain nor pain with either treatment notably affected the cellular composition of the PAG, indicating no major structural PAG alterations. To determine the functional differences of defined cell types between groups, we measured accessible chromatin differences at TF binding site motifs. Motif analysis uncovered several cell-specific TF activity differences that may be indicative of an analgesic mechanism of action in the brain. Specifically, in oligos, we observed an increase in the activity of SOX family TFs that was restored to normal with analgesic treatments. In neurons, we observed restoration of SP/KLF family transcription factors with analgesic treatment. Together, these findings indicate that the ApAP/SRP-001 analgesic treatments modulate crucial functions of these TFs within pain-relevant cell types, including cell recruitment, myelin integrity maintenance, and survival maintenance. We also observed analgesic specialized actions in AP-1 family TFs and TFEB/3 in neurons indicative of anti-inflammation and antinociception induced by analgesic treatments. Notably, the TF activity differences in neurons were independent of gene expression of TFs measured by GEX data, indicating a strong epigenetic response by analgesic treatment, which was also measured by DA analysis, revealing several differences in chromatin accessibility in CFA compared to Vehicle conditions, as well as in treatment groups compared to CFA. To highlight potential antinociceptive effects on neurons at the genome level, DEGs and cell–cell interactions determined by LRP analysis revealed synaptic integrity disruption through Neuregulin-Neurexin signaling caused by CFA pain and recovered with analgesic treatment.

In summation, SRP-001 and ApAP function similarly at both the genetic and epigenetic levels in the midbrain PAG region in the context of CFA-induced pain. These medications support recovery mechanisms important to pro-survival, anti-inflammation, and cell maintenance, all relevant to their analgesic effect. We observe these changes related to pain modulation in both SRP-001 and ApAP, but SRP-001 offers increased safety by preventing liver damage, as previously described (Bazan et al., 2024). The metabolic pathway differences between SRP-001 and ApAP lead to the formation of secondary metabolites, a non-toxic one with SRP-001 and a toxic one with ApAP. The similar analgesic and neurobiological functionality, coupled with the non-hepatotoxicity of SRP-001, demonstrates favorability of SRP-001 over ApAP for pain management.

Our previous findings from the single-cell RNA-seq (Bazan et al., 2024) of the PAG revealed that both SRP-001 and ApAP modulate pain-related gene expression, particularly within the endocannabinoid system and mechanical nociception pathways. Both compounds downregulate FAAH and related genes (e.g., CORO2A, RPL7L1), enhancing levels of antinociceptive endocannabinoids like anandamide (AEA). They also influence genes involved in 2-AG signaling, cannabinoid receptors (CNR1/2), TRPV4, and voltage-gated calcium channels.

ApAP, via its metabolite AM404, activates TRPV1 on glutamatergic neurons in the ventrolateral PAG (vlPAG), enhancing glutamate release and mGlu5 receptor activation (Eric Omo Irinmwinuwa, Prince Chiazor Unekwe, Emmanuel Ugochukwu Egba, Emmanuel Chigemezu Metu, Njoku Charles Cherech. Acetaminophen: Ancient drug with a novel analgesic mechanism of action. World J. Adv. Res. Rev. 16, 580–589, 2022, Mallet et al., 2023, Högestätt et al., 2005, Singh and Kaur, 2020, Barrière et al., 2020). Beyond traditional calcium and sodium channels, potassium channels (K-ATP, KCa, K2P) are emerging as novel analgesic targets in the PAG (Zemel et al., 2018, Beich et al., 2025, Gada and Plant, 2019). SRP-001 broadly modulates ion channels (TRPV1, TRPV4, ASIC3, TRPA1, KCNA1, KCNT1), suggesting a wide-ranging inhibitory effect on nociceptive signaling. TRPV1 is a critical central and peripheral pain target, with research highlighting its dual function in sensing and modulating nociceptive signals within the PAG-Rostral Ventromedial Medulla pathway (Eric Omo Irinmwinuwa, Prince Chiazor Unekwe, Emmanuel Ugochukwu Egba, Emmanuel Chigemezu Metu, Njoku Charles Cherech. Acetaminophen: Ancient drug with a novel analgesic mechanism of action. World J. Adv. Res. Rev. 16, 580–589, 2022, Barrière et al., 2020, Mallet, 2010, Ohashi and Kohno, 2020).

ATAC-seq data revealed novel cell-specific regulatory epigenomic responses to pain and analgesia. In oligodendrocytes, CFA-induced pain increased SOX transcription factor (TF) activity, which was normalized by both drugs, suggesting restoration of myelination and anti-inflammatory functions. In neurons, CFA reduced SP/KLF TF activity, which was restored by SRP-001 and ApAP, indicating recovery of pro-survival and neurite growth pathways. Both drugs also suppressed AP-1 (FOS, JUN) and TFEB/3 TF activity, pointing to epigenetic anti-inflammatory effects.

Cell-type analysis showed CFA increased oligodendrocytes, while SRP-001 and ApAP increased OPCs and microglia. Neuronal shifts included reduced glutamatergic and increased GABAergic populations. Pain disrupted Neurexin-Neuroligin (Nrxn-Nlgn) synaptic signaling, especially Nrxn3-Nlgn1, which was restored by both treatments more robustly by SRP-001, highlighting its potential for long-term synaptic and neural health.

Differential accessibility analysis showed SRP-001 had a stronger epigenomic impact, particularly in GABAergic neurons, underscoring its potent engagement in pain-relief pathways. These findings reveal distinct, cell-specific genomic and epigenomic mechanisms by which SRP-001 and ApAP modulate pain, offering new insights into central analgesia and synaptic resilience.

### Limitations and future directions

A limitation of the current study, particularly concerning our ATAC-seq and RNA-seq analyses, is the use of one biological replicate per condition. While this approach allowed us to generate initial exploratory insights into the genomic and epigenomic landscape following analgesic treatment in a cell-specific manner, it inherently limits our ability to assess inter-sample variability and definitively identify statistically significant differentially expressed genes or accessible chromatin regions. The rationale behind this experimental design was primarily driven by the scarcity of the cell-specific material obtained from the PAG region, the high cost associated with cell-specific sequencing, and the preliminary exploration of these analyses aimed at identifying broad cell-specific transcription patterns and chromatin accessibility landscapes. We acknowledge that future studies employing a larger number of biological replicates will be essential to validate these findings, perform more robust statistical analyses, and thoroughly characterize the extent of genomic and epigenomic modulation. Nevertheless, the present data provide valuable initial indications of cell-specific molecular changes within the PAG region, guiding more powerful future investigations. In addition, we noted that several recent studies have successfully employed n = 1 biological replicate designs in similar contexts using 10xGenomics Multiome assays, with limited biological replicates by employing aggregated gene expression and chromatin accessibility data across all nuclei of a given cell type within a single sample (Gill et al., 2025, Conklin, 2022, Guyer, 2023, Joint RNA and ATAC analysis: 10x multiomic, Marand et al., 2021, Li et al., 2021, Stuart et al., 2021).

There is a notable difference in cell count between conditions, leading to slight differences in statistical power between conditions. However, this does not affect expression or accessibility results as they underwent correction by scaling and normalization methods. This study only includes male rodents and does not address sex differences in genetic or epigenetic response to pain, pain signaling, or analgesic treatment. This study uses RNA and ATAC information to make inferences about the functionality of treatments based on genome and epigenome activity. This assay does not measure protein abundance or function, and results from this study will be validated in the future with functional protein assays. Furthermore, cell–cell interactions using NeuronChat are based on predictions drawn from the expression of ligands and receptors within clusters but are not a direct measure of communication. This will also be validated in the future with functional protein assays.

## Materials and methods

### Experimental design: objectives and design of the study

To observe how treatment with SRP-001 or ApAP following pain affected epigenetic profiling in the PAG, we employed single-cell Multiome. Through data analysis, we planned to determine cell-specific epigenetic modulation that was indicative of potential mechanisms of action of the analgesic treatments.

We recently developed a library of 2-(benzenesulfonamide)-N-(4-hydroxyphenyl) acetamide analgesics to identify non-hepatotoxic analogs of ApAP (Bazan et al., 2020). Among these, SRP-001 was synthesized using high-quality, commercially available analytical reagents without additional purification. Melting points were determined using open capillary tubes on a Stuart Scientific SMP3 apparatus. NMR spectra (1H and 13C) were recorded at room temperature using Mercury VX-300, Bruker BioSpin GmbH 400 MHz, or Varian Unity 500 MHz spectrometers. Chemical shifts are reported in ppm (δ) relative to TMS, with coupling constants (J) expressed in hertz (Hz). Signals are characterized as follows: s for singlet, d for doublet, t for triplet, br for broad, and m for multiplet. NMR FIDs were analyzed using Mestrenova 12.0.4 software. Product purity was assessed through chromatographic analysis on an Agilent 1200 system with a diode array detector and Agilent 1100 MSD-Q mass detector (C18 Luna column, 100 mm × 4.6 mm × 3 µm). The mobile phase consisted of water with 0.1 % formic acid (A) and methanol with 0.1 % formic acid (B), with an elution gradient from 5 % B to 100 % B over 20 min at a flow rate of 1 mL/min (split 1:2 for MS detection). UV wavelengths used for detection were 214 and 254 nm, and mass detection was conducted in the range of 50–1000 *m*/*z*. N-(4-Hydroxyphenyl)-2-(1,1,3-trioxo-1,2-benzothiazol-2-yl) acetamide 1 was synthesized following the method described (Vaccarino et al., 2007).

*Synthesis of N, N-diethyl-2-[[2-(4-hydroxyanilino)-2-oxo-ethyl]sulfamoyl]benzamide (SRP-001):*

To N-(4-hydroxyphenyl)-2-(1,1,3-trioxo-1,2-benzothiazol-2-yl) acetamide 1 (0.165 g, 0.496 mmol), a solution of diethylamine (0.154 mL, 1.5 mmol) in acetonitrile (3 mL) was added. The mixture was refluxed for 16 h. Evaporation under reduced pressure gave a brown residue, which was purified by chromatography [silica gel60 F254, 70–200 mm, ethyl acetate:hexane (6:4)], followed by crystallization from ethyl acetate:hexane, yielding SRP-001 as a white solid (0.132 g, 66 %). mp 171–172 °C; 1H NMR (400 MHz; DMSO‑d6; Me4Si) δ (ppm): 9.69 (s, 1H), 9.18 (s, 1H), 7.91 (dd, *J* = 7.6, 1.5 Hz, 1H), 7.73–7.54 (m, 2H), 7.46 (brs, 1H), 7.43 (dd, *J* = 7.4, 1.5 Hz, 1H), 7.22 (d, *J* = 8.8 Hz, 2H), 6.64 (d, *J* = 8.8 Hz, 2H), 3.77–3.46 (m, 3H), 3.38–3.28 (m, 1H), 3.16–2.95 (m, 2H), 1.17 (t, *J* = 7.0 Hz, 3H), 1.02 (t, *J* = 7.1 Hz, 3H); 13C NMR (101 MHz, DMSO‑d6) δ (ppm): 168.4, 165.4, 153.5, 136.3, 135.3, 132.8, 130.0, 129.3, 128.6, 127.3, 121.0 (2C), 115.0 (2C), 45.6, 42.7, 38.3, 13.1, 11.9; purity by HPLC 99.1 %; MS (ESI+) (*m*/*z*) 406.20 (MH+). Anal. Calcd. for C19H23N3O5S: %C 56.28, %H 5.72, %N 10.36, %S 7.91. Found: %C 56.73, %H 5.85, %N 10.56, %S 8.17.

### Animal models

**Fig. 5:**
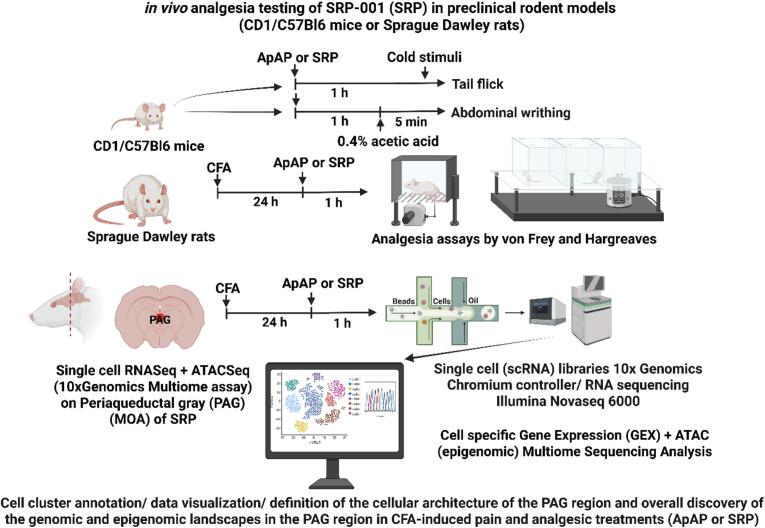
Graphical abstract. Detailed methodology of *in vivo* analgesic testing in preclinical rodent models showing overall discovery of the mechanism of action (MOA) of SRP-001 and ApAP genetic and epigenetic landscapes in the PAG region in CFA-induced pain and analgesic treatments. The experiments for the *in vivo* analgesia were thoroughly presented in our recent publication (Bazan et al., 2024).

All animal protocols and procedures were conducted in accordance with the pre-approved guidelines of the Institutional Animal Care and Use Committee (IACUC) at Louisiana State University Health Sciences Center (LSUHSC), New Orleans. The experiments received approval under IACUC protocol #3739. *In vivo* antinociception assessments were performed using two mouse strains: CD1 and C57BL/6, as well as Sprague-Dawley rats. All laboratory rodents were obtained from Charles River and were acclimated to the LSUHSC New Orleans Neuroscience Center of Excellence vivarium for a minimum of seven days prior to the start of the experimental protocols. The animals were maintained on a 12-hour light/dark cycle with access to food and water available *ad libitum*. For the multiome experiments, we used Sprague Dawley rats that were injected with Complete Freund’s adjuvant (CFA) or Saline and later ApAP or SRP-001 were administered per os. The left hind paws of Sprague-Dawley rats were injected with 150 µl of 50 % CFA, freshly diluted with sterile saline on the day of administration, to induce significant long-term inflammatory pain and mechanical hyperalgesia, assessed using eVF. Following this, the rats were administered either ApAP or SRP-001 at a dose of 100 mg/kg. After one hour, the rats were euthanized, and the PAG was dissected from their flash-frozen brains. A graphical representation highlighting the detailed methodology of the experimental outline is provided in Fig. 5 (Graphical Abstract).

We previously comprehensively reported (Bazan et al., 2024), detailed analgesic efficacy data across multiple preclinical pain models (CFA-induced inflammatory pain with von Frey and Hargreaves assays in Sprague-Dawley rats; tail-flick and abdominal writhing assays in CD1 and C57BL/6 mice) – demonstrated in Fig. 2, Figs. S3–S7, and Table S1 in that publication). In this current study, Sprague-Dawley rats were specifically selected because they demonstrated robust, reproducible, and validated analgesic responses in prior experiments using von Frey and Hargreaves assays. Leveraging the same species and strain allows us to precisely connect behavioral outcomes to the novel genomic and epigenomic mechanisms investigated here via integrated single-cell RNA and ATAC sequencing. Notably, no significant differences in analgesic efficacy of SRP-001 were observed across rat and mouse models or between the different rodent strains tested in prior studies, thus justifying our focused mechanistic approach using Sprague-Dawley rats in the current investigation.

For Hargreaves and von-Frey assays:

*Young male rats*

In this cohort, n = 40 male Sprague–Dawley rats (2 months) were used, and two different doses of SRP-001 oral nanosuspension and ApAP (32 and 100 mg/kg) were compared to a vehicle control.

*Young female rats*

In this cohort, n = 40 female Sprague–Dawley rats (2 months) were used, and two different doses of SRP-001.

oral nanosuspension and ApAP – 32 and 100 mg/kg were compared to a vehicle control.

*Aged male rats*

In this cohort, n = 20 male Sprague–Dawley rats (20 months) and two different doses of SRP-001 oral nanosuspension and ApAP – 32 and 100 mg/kg were compared to a vehicle control.

For the tail-flick assays:

We used n = 70 aged male mice, n = 90 young male mice, and n = 120 young female mice. Based on the availability of mice at the time of the experiments, different numbers of animals were assigned to different cohorts of experimental animals.

For the abdominal writhing assays:

CD1 (young male and female) or C57BL/6 (aged male) mice were used. We used n = 35 aged male mice, n = 70 young male mice, and n = 70 young female mice.

For the single-cell RNASeq and ATACSeq (10xGenomics multiome assays):

PAG region was dissected from the isolated brains of young male Sprague Dawley rats, n = 1 per condition (Vehicle, CFA, ApAP, and SRP-001).

Sprague Dawley rats were used for these experiments (RNASeq + ATACSeq) as most of the *in vivo* analgesic data was obtained by the von-Frey and Hargreaves assays using these rats, which is a well-established preclinical rodent model for such experiments. Since we were trying to decipher a molecular mechanism of action of analgesia for ApAP and SRP-001 using a powerful single-cell/nuclei RNA sequencing (genetic) and ATAC sequencing (epigenetic) approach, we used the same model, and dosed and harvested the brain to isolate the midbrain PAG region from the same species – Sprague Dawley rats.

Rats and mice showed comparable analgesic effects of both SRP-001 and ApAP. Also, there were no differences in the analgesic effects between the different species and different strains. Our results showed comparable analgesic effects, with SRP-001 being better than ApAP. Although, we would like to point out here that we cannot clearly predict similarities or differences between them, as different *in vivo* analgesic assays were performed in different models – von Frey and Hargreaves in Sprague Dawley rats, while Tail flick and Abdominal writhing assays in CD-1 and C57Bl6j mice.

### Data analysis

Generation of libraries for 10x Genomics Single-Nuclei Multiome ATAC + Gene Expression Sequencing:

For both ATAC-seq and RNA-seq, one biological replicate, n = 1 per condition (Vehicle, CFA, ApAP, and SRP-001), was utilized. To isolate cell nuclei from the midbrain PAG, the tissue was flash-frozen, dissected with a cryotome, minced into small pieces, homogenized, filtered through a 70 μm strainer, and centrifuged. ReadiDrop 7-AAD cell viability dye (Bio-Rad) was added, and live nuclei (7-AAD positive) were sorted using a BioRad Cell Sorter S3e in purity mode. Nuclei were counted with a Nexcelom automated Cellometer using acridine orange/propidium iodide (AOPI) staining, and the nuclei stock concentration was adjusted to target 10,000 nuclei per sample. The samples were bulk transposed, and approximately 16,100 nuclei were loaded into each channel of the microfluidic chip, with GEMs generated using the 10x Chromium controller and reagents from the Chromium Next GEM Single Cell ATAC Kit, 1,000,280 (10x Genomics), following the manufacturer’s instructions. After post-GEM cleanup with Dynabeads and SPRIselect (Beckman Coulter), samples underwent pre-amplification PCR and were then divided for constructing ATAC and GEX libraries. Quality control for the libraries was performed using an Agilent Bioanalyzer 2100 with High Sensitivity DNA chips. After verifying sample traces, each library was normalized based on the average fragment size, and concentration was determined using a Qubit 4.0 fluorometer (Thermo Fisher Scientific). The pooled libraries were subsequently sequenced using Illumina high-output kits on a NovaSeq 6000 platform from Illumina.

### Data processing

Cell Ranger ARC (cellranger-arc) v.2.0.2 from 10x Genomics was utilized to process Chromium Single Cell Multiome ATAC + Gene Expression (GEX) sequencing data. Initially, raw BCL files from the Illumina NovaSeq were demultiplexed into paired-end, gzip-compressed FASTQ files using the default parameters of cellranger-arc mkfastq. Subsequently, the cellranger-arc count function was employed for read alignment, filtering, barcode counting, peak calling, and the quantification of both ATAC and GEX molecules. The rat genome mRatBN7 and its annotated transcriptome served as the reference for alignment. Only confidently mapped reads with valid barcodes, unique molecular identifiers (UMIs), and non-PCR duplicates were retained. The overall sequencing quality was assessed by reviewing the summary metrics provided in the web_summary.html file generated for each sample. Processed datasets from multiple samples were then aggregated using cellranger-arc aggr to normalize input runs to the same median fragments per cell across samples. This tool also facilitated the exploration of the relationship between chromatin accessibility and GEX for each sample, as both ATAC and GEX measurements were obtained from the same cells.

Aggregated filtered feature matrix and fragment outputs for GEX and ATAC from CellRanger pipeline were input into a Seurat (Hao et al., 2024) object in R. Chromatin Assay was annotated using mRatBN7.2 annotation. Quality control was performed by filtering out cells with nCount_ATAC < 500 or > 100000, nucleosome_signal > 2, TSS.enrichment < 0.5, nFeature_RNA < 200 or > 7000, and percent.mt > 10 (Fig. S1). SCTransform normalization was performed on GEX data for clustering. Following PCA, dimensionality reduction was performed using PCA dimensions 1:40 for UMAP and a resolution of 0.8 for clustering. MACS2 (Mistry, et al., 2023) was used to call peaks before ATAC clustering. ATAC normalization was performed in Signac (Stuart et al., 2021) using the term frequency-inverse document frequency by RunTFIDF function and singular value decomposition by RunSVD function for LSI dimensionality reduction. ATAC clustering and UMAP were performed using LSI dimensions 2:30 and resolution of 0.5. Weighted Nearest Neighbor (WNN) integration (Hao et al, 2021) was implemented using PCA and LSI dimensions defined above and a clustering resolution of 0.5 (Fig. S2). Signac was used for Peak Linkage with a score cutoff of 0.03 and ChromVAR (Schep et al., 2017) for transcription factor (TF) analysis using the JASPAR2020 (Fornes et al., 2020) database. The RNA dataset was mapped to the Allen Brain Cell (ABC) Atlas 10x Whole Mouse Brain taxonomy using the tool MapMyCells (RRID:SCR_024672) (MapMyCells (RRID:SCR_024672)) and the Hierarchical Mapping algorithm. Cell annotations were manually adjusted based on the cell types, as seen in Fig. 1A. To better separate specific neuronal types, all annotated neurons were further analyzed as a subset. The same normalization and clustering process as described above was performed using PCA dimensions 1:40 and a resolution of 1.5 for clustering for RNA, LSI dimensions 2:10 and a resolution of 1.5 for clustering for ATAC and integrated using WNN with a resolution of 1.5 for clustering. Neuron types were determined based on MapMyCells output and manually adjusted into two groups based on the expression of Gad1 for GABAergic neurons and Slc17a7 for Glutamatergic neurons.

### Downstream analysis

Differential Expression (DE) analysis was performed using the SCT assay and Seurat’s FindMarkers function between conditions for each cell type with a min.pct of 0.05 (Figs. S3 and S4). Mitochondrial, Gm, and Rik genes were filtered out. Cell-cell interaction analysis was performed using neuronal clusters using NeuronChat (Zhao et al., 2023). RNA data was used for each condition to create NeuronChat objects, and analysis was run using M = 100. Comparative analysis was performed by merging objects. Differential Accessibility (DA) analysis was performed using ATAC assay and Seurat’s FindMarkers function between conditions for each cell type with a min.pct of 0.05. Motif Enrichment analysis was performed using chromVAR assay using row means to determine the average difference. TF Footprint analysis was performed in Signac using the Footprint function for the top motifs of interest determined by Motif Enrichment.

### Statistical methods

Comparative analysis for DA (Data S1), DE (Data S2), and Motif Enrichment (Data S3) was performed using FindMarkers based on non-parametric, Wilcoxon rank sum test. P-values determined from this test were adjusted for multiple testing using Bonferroni correction methods per Seurat.

## CRediT authorship contribution statement

**Hernan A. Bazan:** Writing – original draft, Supervision, Conceptualization. **Brian L. Giles:** Writing – original draft, Visualization, Formal analysis, Data curation. **Surjyadipta Bhattacharjee:** Writing – review & editing, Writing – original draft, Methodology, Formal analysis. **Scott Edwards:** Writing – review & editing, Methodology. **Nicolas G. Bazan:** Writing – review & editing, Supervision, Conceptualization.

## Declaration of competing interest

The authors declare the following financial interests/personal relationships which may be considered as potential competing interests: HAB and NGB are named on a patent assigned to the Board of Supervisors of Louisiana State University and Agricultural and Mechanical College describing the synthesis and characterization of the novel non-hepatotoxic acetaminophen analogs, patent application PCT/US2018/022029, international filing data 12.03.2018; publication date 28.02.2019; which has been nationalized in numerous jurisdictions. HAB and NGB are co-founders of South Rampart Pharma, which has a license and commercial interest in the above patent applications, including a financial interest in the commercial success of SRP-001. They stand to potentially receive financial payments if SRP-001 is commercially successful.

## Data Availability

All sequencing data have been uploaded to GEO under the accession number GSE286589. All analysis was conducted using publicly available tools and packages. Analytical workflow, including specific parameters for analysis, was outlined in the Methods section.
