# Supplementary material for: A non-toxic analgesic elicits cell-specific genomic and epigenomic modulation by targeting the PAG brain region
Source: Neurobiol Pain. 2025 Jul 20;18:100192. doi: 10.1016/j.ynpai.2025.100192 (PMC12312049; doi:10.1016/j.ynpai.2025.100192)
Supplement: Supplementary Data 1 [file mmc1.docx]

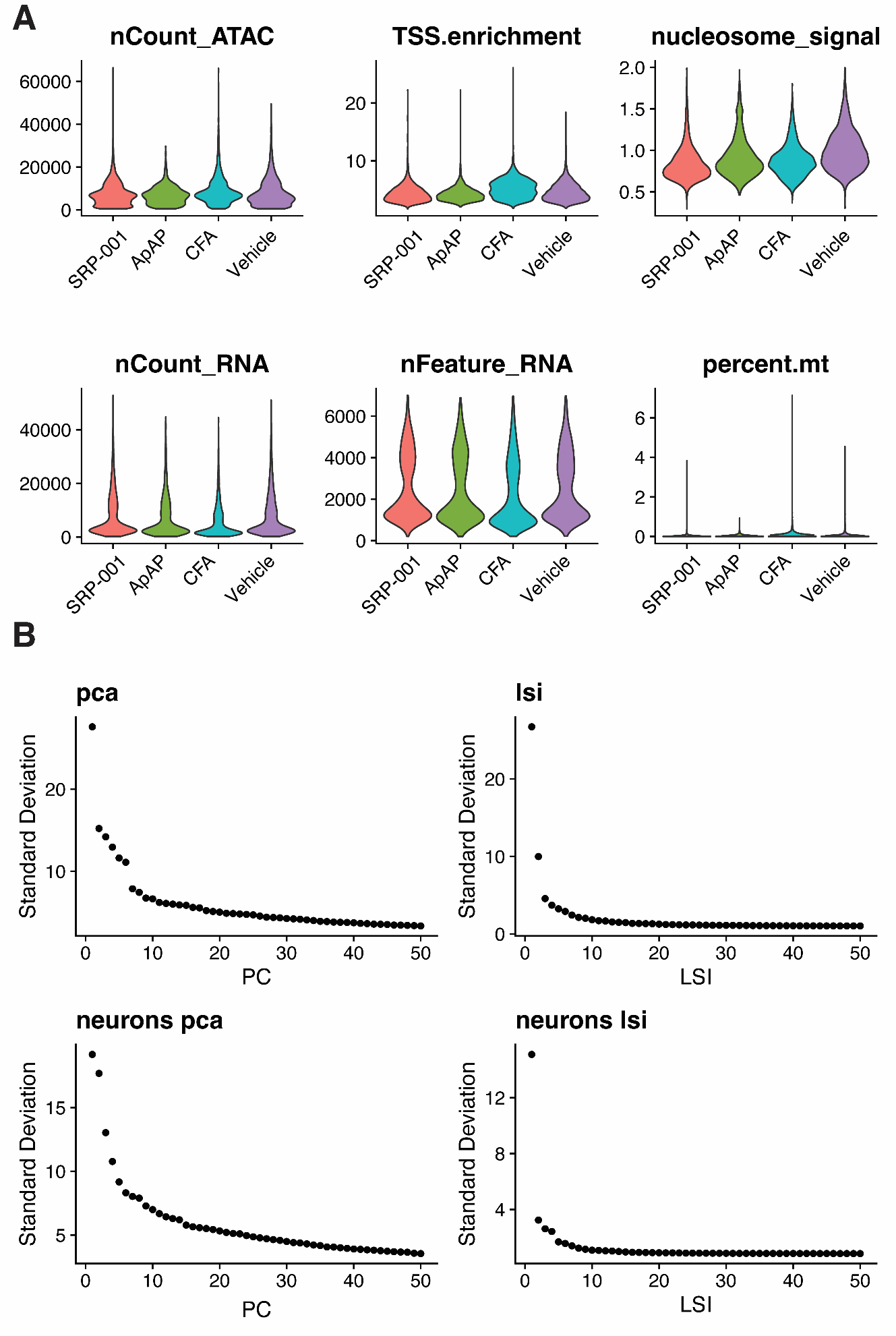


**Suppl. Fig.1 Quality Control and Dimensionality Reduction.** Violin plots displaying quality control features showing similarity between each sample (A). Elbow plots displaying results of principle component analysis (PCA) for GEX data and latent semantic index (LSI) for ATAC data for all cells and neuronal subset (B).


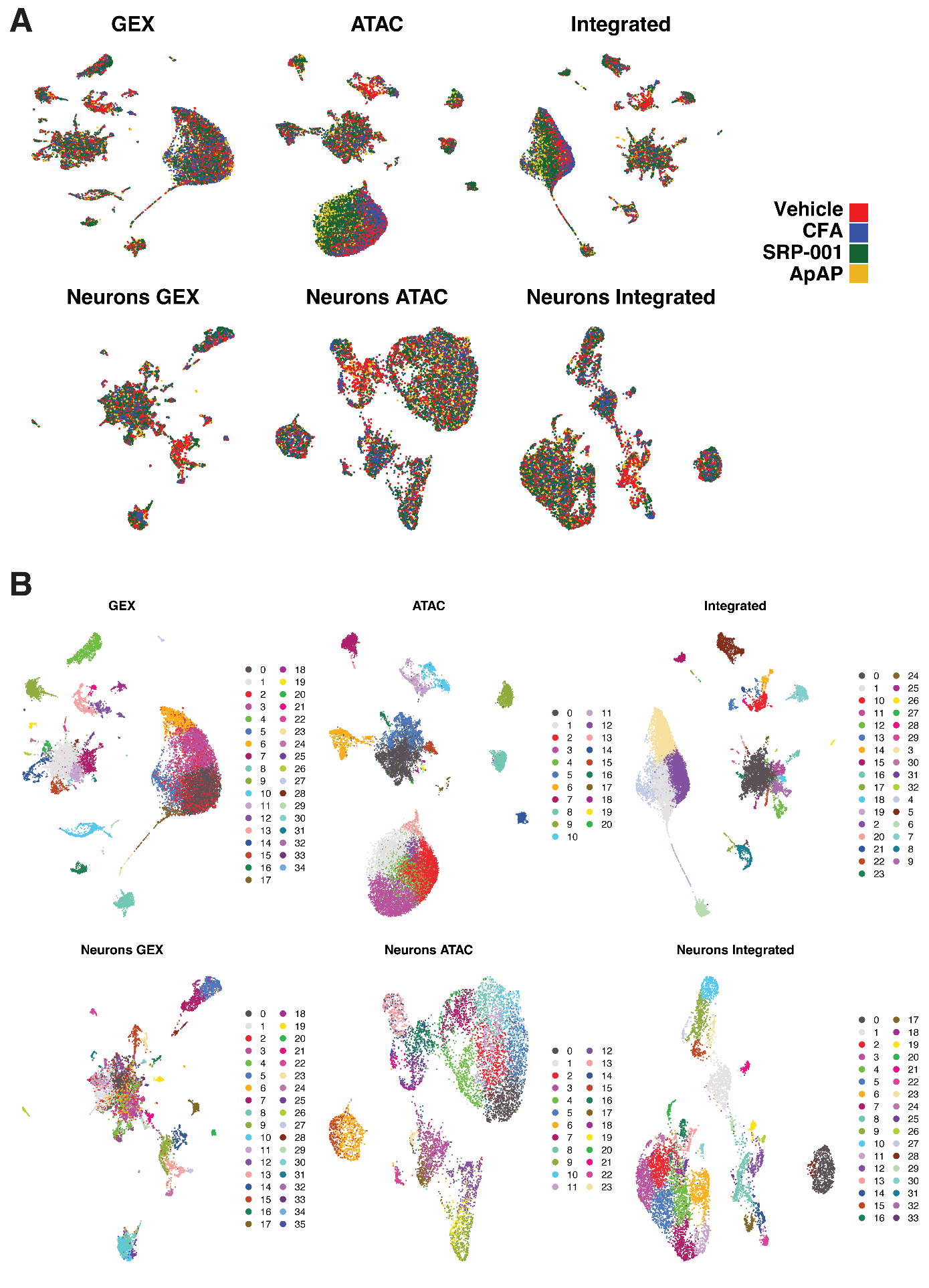


**Suppl. Fig.2 Cell Clustering.** UMAPs displaying clustering results for all cells and neuronal subset separated by GEX, ATAC and WNN integration colored by sample (A) and by Seurat clusters (B).


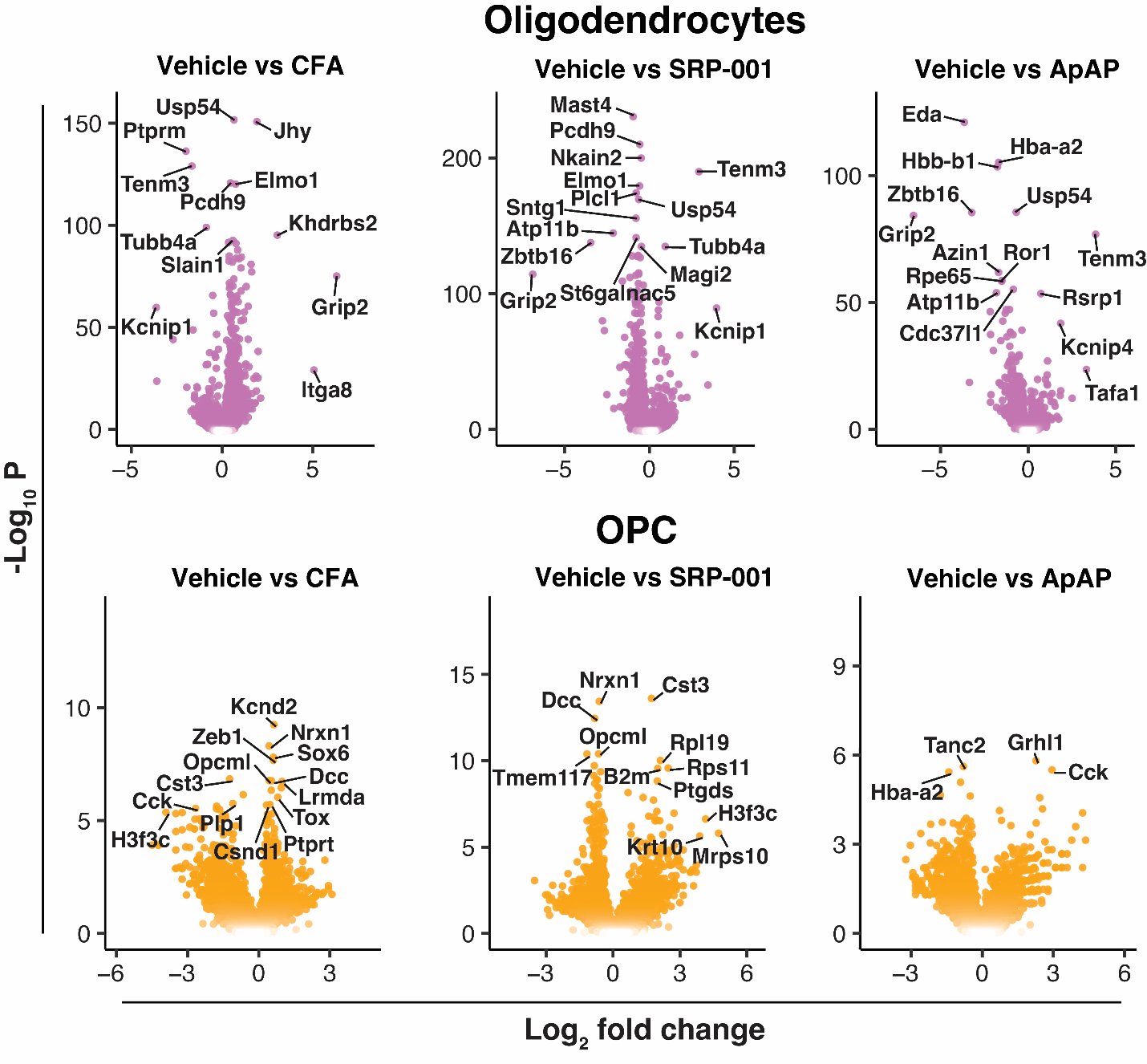


**Suppl. Fig.3 Differential Expression Profile of Oligodendrocytes and OPCs.** Volcano plots displaying top DEGs for listed comparisons. Top significant genes are labeled.


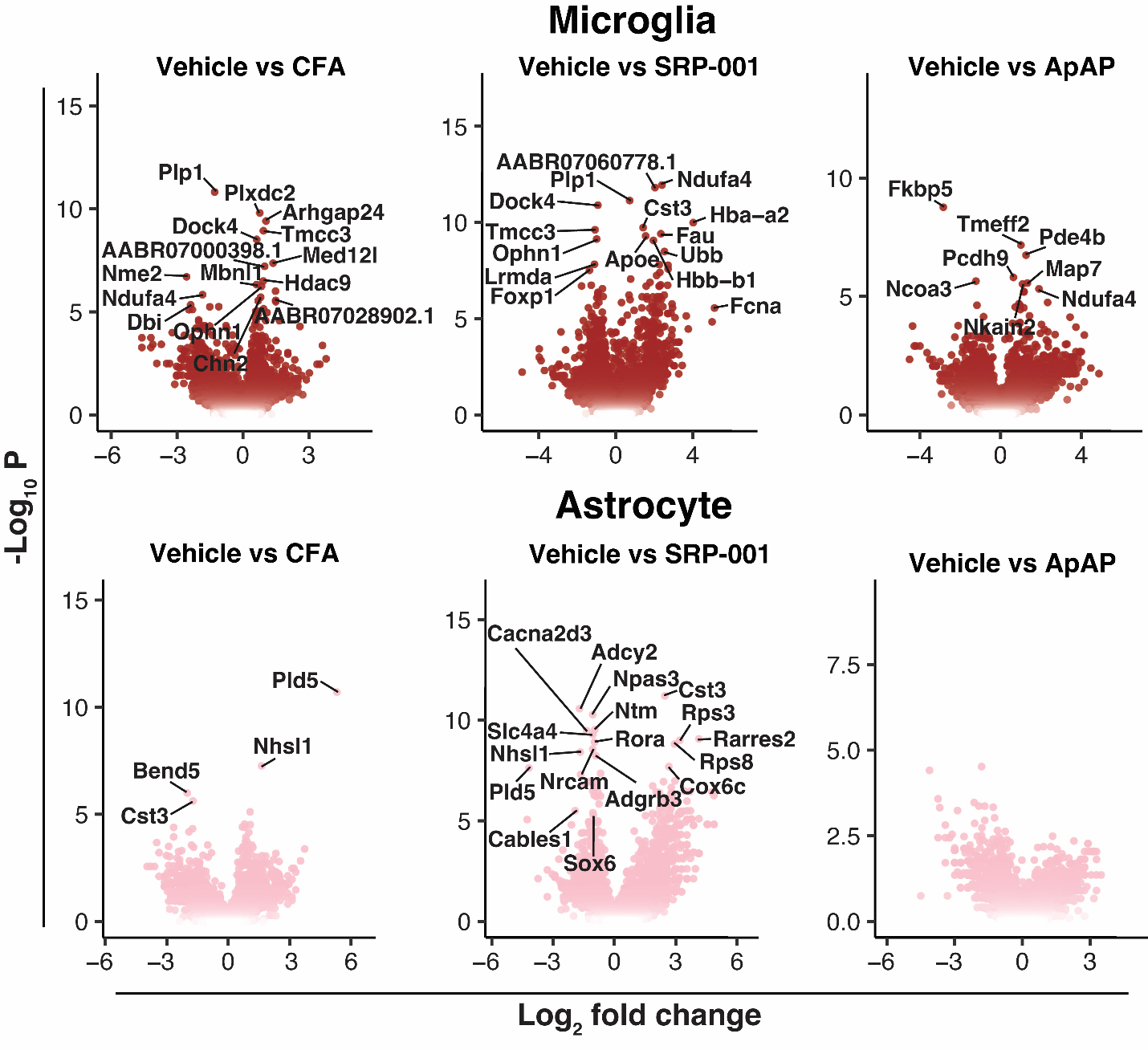


**Suppl. Fig.4 Differential Expression Profile of Microglia and Astrocytes.** Volcano plots displaying top DEGs for listed comparisons. Top significant genes are labeled.


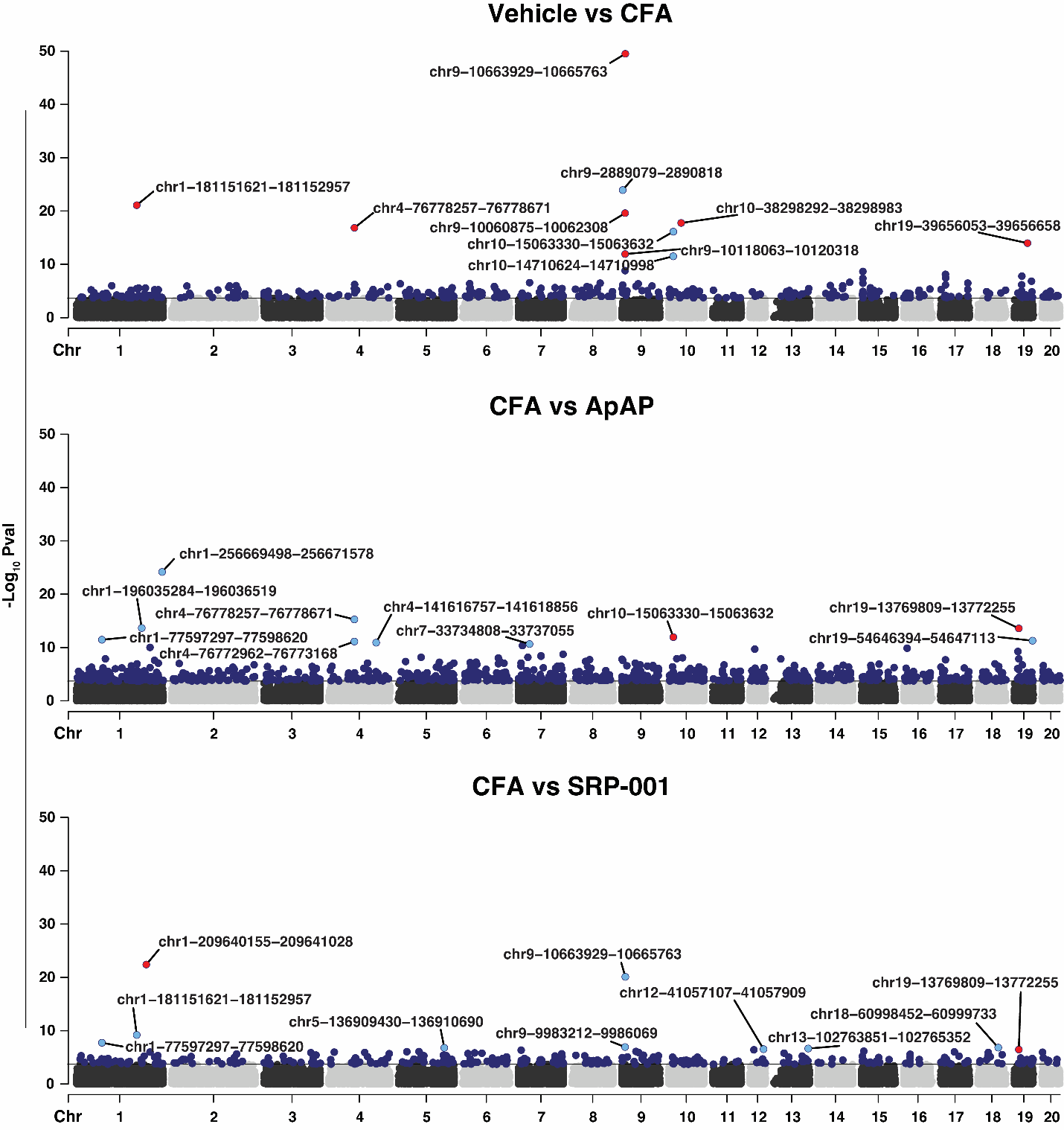


**Suppl. Fig.5 Chromatin Accessibility of Glutamatergic Neurons.** DA results for listed comparisons in Glutamatergic neurons. All significant regions (pval < 0.05) are highlighted by dark blue color. Top few regions in each comparison labeled with red or light blue color for whether they increase or decrease in second condition of comparison.


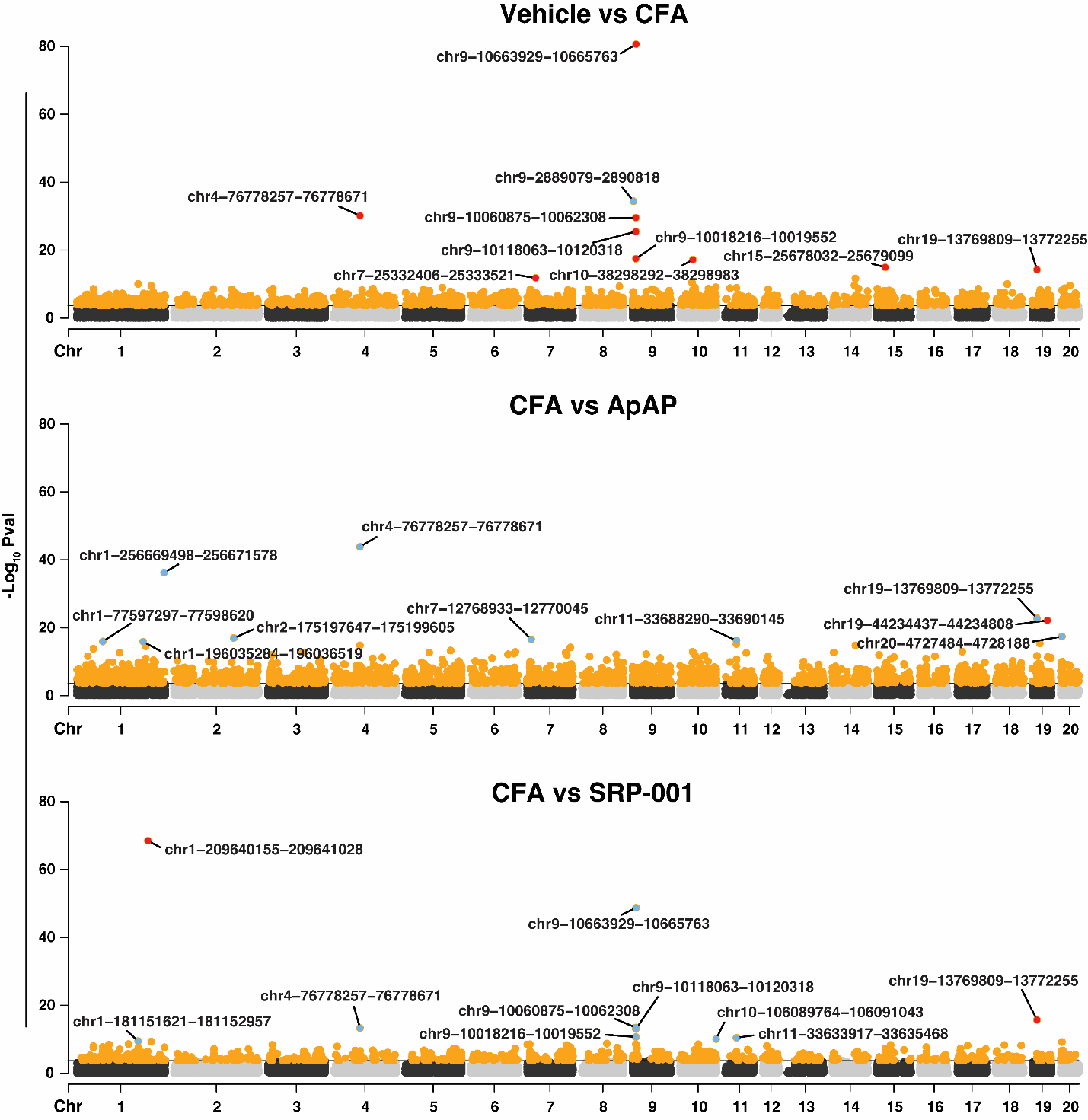


**Suppl. Fig.6 Chromatin Accessibility of GABAergic Neurons.** DA results for listed comparisons in GABAergic neurons. All significant regions (pval < 0.05) are highlighted by yellow color. Top few regions in each comparison labeled with red or light blue color for whether they increase or decrease in second condition of comparison.


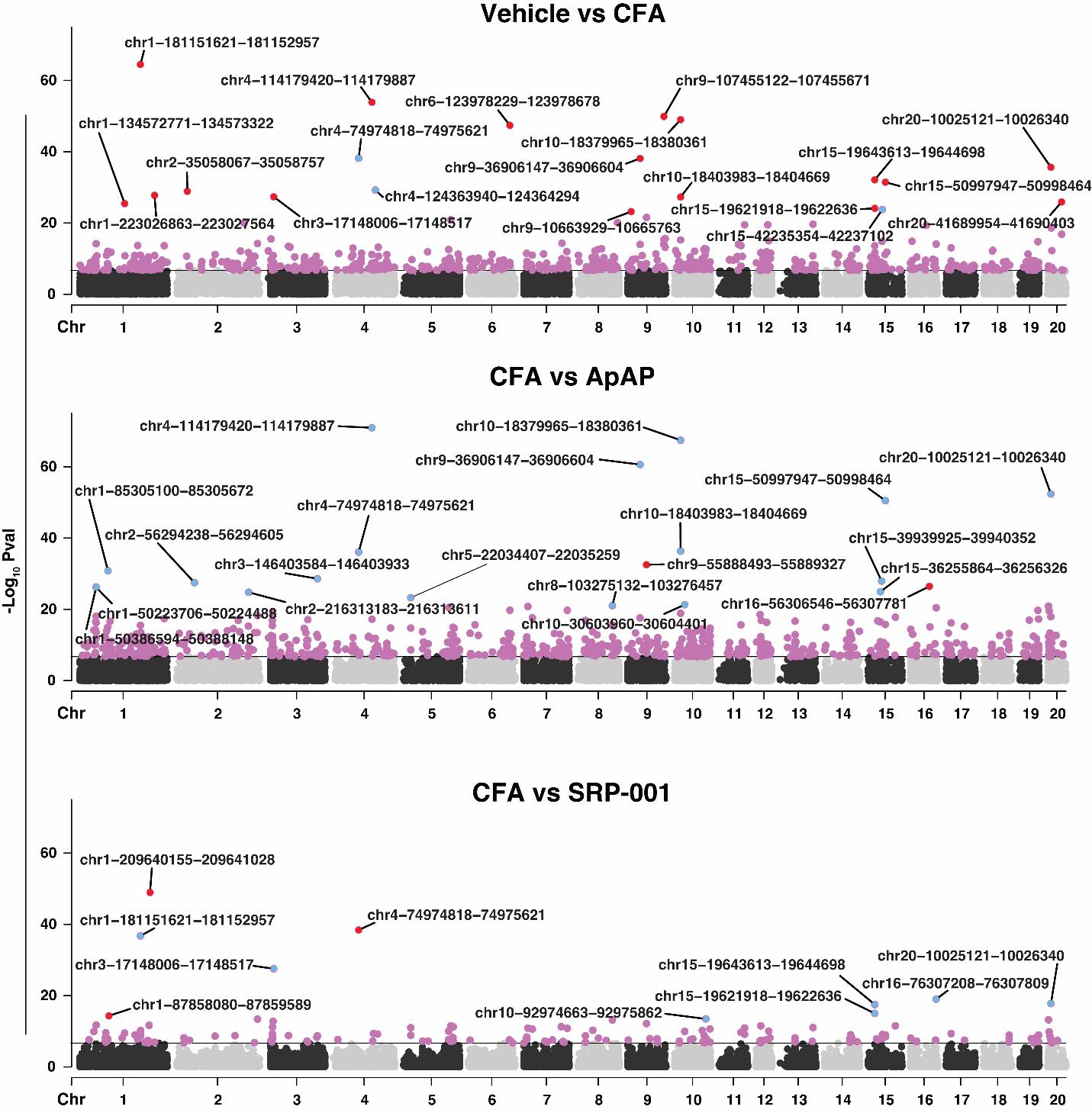


**Suppl. Fig.7 Chromatin Accessibility of Oligos.** DA results for listed comparisons in Oligos. All significant regions (pval < 0.05) are highlighted by purple color. Top few regions in each comparison labeled with red or light blue color for whether they increase or decrease in second condition of comparison.

**Supplementary Tables -**

**Table1 – Cell Types per Condition**

|  | **Vehicle** | **CFA** | **SRP-001** | **ApAP** |
| --- | --- | --- | --- | --- |
| **Neurons** | 2573 | 1900 | 3187 | 828 |
| **Oligo** | 2522 | 2593 | 3875 | 920 |
| **OPC** | 259 | 71 | 324 | 132 |
| **Microglia** | 236 | 120 | 370 | 108 |
| **Astrocytes** | 68 | 38 | 235 | 29 |
| **Total** | 5658 | 4722 | 7991 | 2017 |

**Table2 – Neuron Types per Condition**

|  | **Vehicle** | **CFA** | **SRP-001** | **ApAP** |
| --- | --- | --- | --- | --- |
| **Glut** | 1171 | 774 | 1111 | 245 |
| **GABA** | 1402 | 1126 | 2076 | 583 |
| **Total** | 2573 | 1900 | 3187 | 828 |

All sequencing data has been uploaded to GEO under the accession number GSE286589. All analysis was conducted using publicly available tools and packages. Analytical workflow, including specific parameters for analysis, was outlined in the Methods section. Under the GEO under the accession number GSE286589, data are organized as DE Results, DA Results, and Motif Enrichment Results. All these results are in zipped folders as described below. The zipped folders can be shared upon request to the corresponding authors.

**Zipped Folders –**

Data 1 - DE Results.zip

Data enclosed consists of DE results from GEX data. Pct1 and Pct2 correspond to the order of condition in the title.

Data 2 - DA Results.zip

Data enclosed consists of DA results from ATAC data. Pct1 and Pct2 correspond to the order of condition in the title.

Data 3 - Motif Enrichment Results.zip

Data enclosed consists of ChromVAR results for motif enrichment. Pct1 and Pct2 correspond to the order of condition in the title.
